# Proteomic analysis of combined IGF1 receptor targeted therapy and chemotherapy identifies signatures associated with survival in breast cancer patients

**DOI:** 10.18632/oncotarget.27566

**Published:** 2020-04-28

**Authors:** Tali Sinai-Livne, Metsada Pasmanik-Chor, Zoya Cohen, Ilan Tsarfaty, Haim Werner, Raanan Berger

**Affiliations:** ^1^ Department of Human Molecular Genetics and Biochemistry, Sackler School of Medicine, Tel Aviv University, Tel Aviv 69978, Israel; ^2^ Bioinformatics Unit, George Wise Faculty of Life Sciences, Tel Aviv University, Tel Aviv 69978, Israel; ^3^ Institute of Oncology, Chaim Sheba Medical Center, Tel Hashomer 52620, Israel; ^4^ Department of Clinical Microbiology and Immunology, Sackler School of Medicine, Tel Aviv University, Tel Aviv 69978, Israel; ^5^ Yoran Institute for Human Genome Research, Tel Aviv University, Tel Aviv 69978, Israel

**Keywords:** insulin-like growth factor-1 (IGF1), IGF1 receptor (IGF1R), targeted therapy, breast cancer, proteomic analysis

## Abstract

Clinical, epidemiological and experimental data identified the insulin-like growth factor-1 receptor (IGF1R) as a candidate therapeutic target in oncology. While this paradigm is based on well-established biological facts, including the potent anti-apoptotic and cell survival capabilities of the receptor, most Phase III clinical trials designed to target the IGF1R led to disappointing results. The present study was aimed at evaluating the hypothesis that combined treatment composed of selective IGF1R inhibitor along with classical chemotherapy might be more effective than individual monotherapies in breast cancer treatment. Analyses included comprehensive measurements of the synergism achieved by various combination regimens using the *CompuSyn* software. In addition, proteomic analyses were conducted to identify the proteins involved in the synergistic killing effect at a global level. Data presented here demonstrates that co-treatment of IGF1R inhibitor along with chemotherapeutic drugs markedly improves the therapeutic efficiency in breast cancer cells. Of clinical relevance, our analyses indicate that high IGF1R baseline expression may serve as a predictive biomarker for IGF1R targeted therapy. In addition, we identified a ten-genes signature with potential predictive value. In conclusion, the use of a series of bioinformatics tools shed light on some of the biological pathways that might be responsible for synergysm in cancer therapy.

## INTRODUCTION

Breast cancer is the most frequently diagnosed cancer worldwide. Breast cancer causes the greatest number of cancer-related deaths among women as one in eight to ten women will develop the disease during their lifetime [1]. According to the World Health Organization, breast cancer accounts for 25% of all female cancers and 12% of all cancers [2, 3]. The insulin-like growth factors (IGF1, IGF2) are a family of mitogenic polypeptides with important regulatory roles in multiple physiological processes, including metabolic, nutritional and endocrine events [4–6]. Deregulated expression or activity of IGF family members has been linked to several pathologies, ranging from growth deficits to cancer development [7–10]. The IGF system has an important role in the development and maturation of the mammary gland as well as in breast cancer initiation and progression [11, 12]. The IGF1 receptor (IGF1R), which mediates the biological actions of IGF1 and IGF2, exhibits potent antiapoptotic and cell-survival activities and is regarded as a key player in breast cancer etiology [13–16].

The IGF1 axis and, particularly, the IGF1R have emerged in recent years as promising therapeutic targets in oncology [17–21]. Empirical support to this view was provided by preclinical studies showing that IGF1R *hyper*activation constitutes a fundamental prerequisite for cancer development [13, 22, 23]. Furthermore, the *IGF1R* gene is overexpressed by most tumors, hence conferring a survival advantage to malignantly transformed cells. Despite a strong preclinical rationale, the vast majority of Phase III studies using IGF1R monoclonal antibodies or selective IGF1R inhibitors in unselected patients led to disappointing results [24]. As a consequence of these negative outcomes there is an urgent need to identify molecular predictors of sensitivity to IGF1R inhibitors that may help in selecting potential responders. Discovery of novel biomarkers is expected to have major translational implications [25]. Furthermore, it is of cardinal relevance to generate evidence-based proof of potential benefits of multi-targeted cancer therapy as compared to IGF1R-directed monotherapy.

Extensive molecular profiling revealed that a number of components of the IGF signaling pathway, including insulin receptor substrate-2 (IRS2) and IGF-binding protein-5 (IGFBP5) among others, play key roles in determining the sensitivity of cancer cells to a humanized IGF1R antibody [26]. Similarly, IGF1R expression levels and activation (*i. e*., phosphorylation) status, as well as additional downstream mediators, might also help in selecting patients for targeted therapy [12, 27]. The present study was aimed at: (1) evaluating the efficacy of IGF1R inhibition as monotherapy in comparison to combination treatment with chemotherapy in breast cancer cells; (2) assessing the potential synergism achieved by combination therapy; and (3) characterizing this combined approach from a proteomic/bioinformatics perspective. In addition, public databases of breast cancer patients were analyzed in order to address the impact of IGF1R expression on survival and to identify potential ‘signatures’ associated with improved survival. Data obtained revealed that combined IGF1R-directed therapy along with classical chemotherapy led to a synergistic killing effect. Global proteomic analyses identified a number of proteins that may play mechanistic roles in the synergistic cell growth inhibition. Furthermore, clinical databases mining revealed that high IGF1R levels were correlated with better survival probability. Finally, our analyses identified a ten-genes signature with potential predictive value. Future studies will assess the clinical relevance of our data.

## RESULTS

### Effects of IGF1R inhibitors on MCF7 cells viability

Initial experiments were aimed at identifying an effective IGF1R inhibitor suitable for our experimental *in vitr*o conditions. To this end, four different IGF1R inhibitors were assessed on MCF7 cells. Two of the inhibitors (MK-0646 and A12) were humanized IgG1 monoclonal antibodies that bind to the extracellular domain of IGF1R, thus preventing ligand-induced receptor activation. The other two compounds were selective small MW IGF1R tyrosine kinase inhibitors (AEW541 and AG1024). Results of XTT assays indicate that AEW541 reduced cell viability by 50% after 72 h (Supplementary Figure 1A). Treatments with MK-0646, A12 or AG1024 had no major inhibitory effects, probably as a result of the fact that experiments were conducted using serum-containing media. We assume that the particular mechanism of action of AEW541 (*i. e*., reversible, ATP-competitive inhibition of IGF1R but not insulin receptor) might, probably, explain the fact that only this compound was capable of reducing viability under our experimental conditions. While, as expected, antibodies MK-0646 and A12 reduced total IGF1R protein expression levels at 24 and 48 h, small MW compounds AEW541 and AG1024 had no effect on IGF1R protein levels (Supplementary Figure 1B). Based on these results, the following experiments were conducted using the AEW541 inhibitor.

### Effect of AEW541 on IGF1-mediated signaling

**Figure 1:**
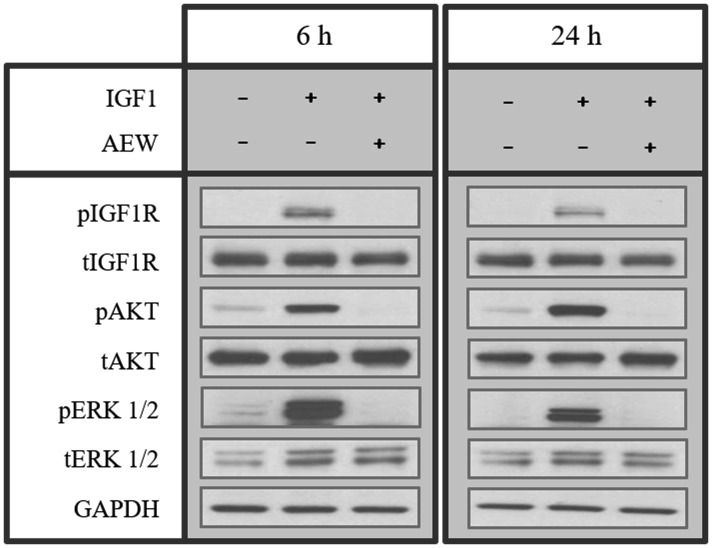
Effect of AEW541 on IGF1-mediated signaling. MCF7 cells were treated with AEW541 (2 μM) for 6 h and 24 h, followed by IGF1 (50 ng/ml) treatment during the last 10 min of the incubation period. Cells were then lysed and the levels of phosphorylated and total IGF1R, AKT and ERK1/2 proteins were measured by Western blots. Equal loading was confirmed by GAPDH loading.

To further assess the impact of IGF1R inhibition on IGF1-mediated signaling, MCF7 cells were treated with AEW541 for 6 or 24 h, in the presence of IGF1 during the last 10 min of the incubation period. The IGF1 dose employed (50 ng/ml) is regarded as a physiological concentration. As shown in Figure 1, AEW541 abolished the IGF1-stimulated IGF1R phosphorylation at both time points. Furthermore, AEW541 prevented activation of the AKT and ERK1/2 cytoplasmic mediators.

### Effect of combined AEW541 and chemotherapy treatment

**Figure 2:**
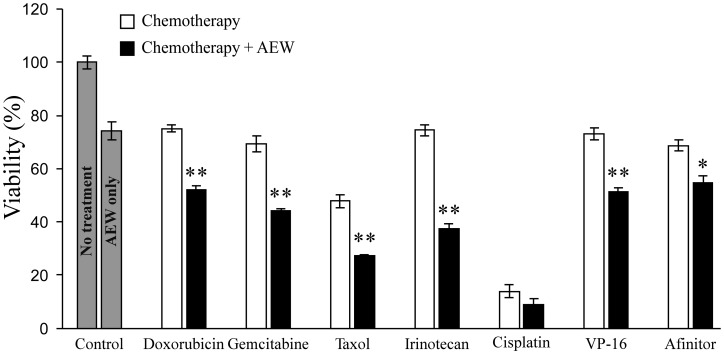
Effect of AEW541 in combination with anti-cancer drugs on MCF7 cell viability. MCF7 cells were seeded in 96- well plates s at a density of 3 × 10^3^ cells per well. After 24 h, cells were treated with a panel of anti-cancer agents, alone or in combination with AEW541 (3 μM). After 48 h, cell viability was measured using XTT assays. Bars represent mean ± SEM of three independent experiments. Statistical analysis was done using Student’s *t*-test (^**^ *p <* 0.01 or ^*^ *p <* 0.05 drug *versus* drug + AEW541 or untreated cells *versus* AEW541).

Next, the effect of combined AEW541 treatment with a panel of seven anti-cancer drugs on cell viability was examined. The following drugs were used in these experiments: doxorubicin, gemcitabine, taxol, irinotecan, cisplatin, VP-16 or the biological drug Afinitor (a PARP inhibitor). AEW541 as a single agent caused a 26% decrease in cell viability compared to untreated cells (Figure 2). The combined treatment of AEW541 with each one of the drugs, except cisplatin, caused a significant reduction in cell viability (in comparison with the single-agent treatment), suggesting a potential synergistic effect of the combination therapies.

### Analysis of synergistic cell growth inhibition

**Figure 3:**
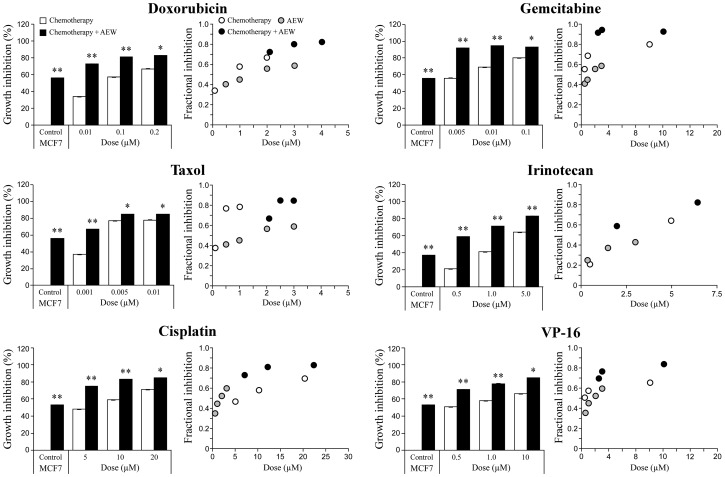
Synergistic killing effect of AEW541 in combination with chemotherapeutic agents. MCF7 cells were seeded in 96-well plates at a density of 3 × 10^3^ cells per well. After 24 h, cells were treated with a panel of six chemotherapeutic agents, alone or in combination with AEW541. After 72 h, cell viability was measured using XTT assays. Bara represents the mean ± SEM of three independent experiments. Statistical analysis was performed by using unpaired Student’s t-test (^**^ *p <* 0.01 or ^*^ *p <* 0.05 drug *versus* drug + AEW541 or untreated cells *versus* AEW541). The CI values (combination index) were determined by the *Compusyne* software for all of the combined treatments.

To select an anti-cancer drug to further dissect the synergistic effect of the combined treatment, the dose-response curves of these agents on growth inhibition, as single agents or in combination with AEW541, were examined. In comparison to cell viability, which is defined as the percentage of live cells in a whole sample, growth inhibition is the specific reduction in growth of tumor cells upon treatment. To this end, MCF7 cells were treated with the various drugs for 72 h and then proliferation was measured by XTT assays. The *Compusyne* software was employed to evaluate the synergistic effect of the combined treatments and the intensity of the synergy was expressed in combination index (CI) values. Data demonstrate that combination of AEW541 with each of the different chemotherapeutic drugs enhanced growth inhibition in comparison with the single-agent treatment (Figure 3 and Table 1). While a synergistic effect was seen with all six combinations, combination of IGF1R targeting with gemcitabine generated the strongest effect and was therefore selected for further analyses.

**Table 1:** CI values for the synergistic effect of AEW541 and chemotherapeutic drugs

Cells	Drug	Drug Dose (uM × 10)	Dose AEW(uM)	Effect	Cl	Synergism
		0.5		0.59	0.24	Strong
	rinotecan	1.0	1.5	0.71	0.17	Strong
		5.0		0.83	0.31	Synergism
		0.5		0.92	0.01	Very strong
	Gemcitabine	1.0		0.95	0.00	Very strong
		10.0		0.93	0.04	Very strong
		0.1		0.67	0.56	Synergism
	Taxol	0.5		0.85	0.40	Synergism
MCF7		1.0		0.85	0.77	Moderate
		5.0		0.75	0.37	Synergism
	Cisplatin	10.0	2.0	0.83	0.26	Strong
		20.0		0.85	0.36	Synergism
		0.5		0.71	0.29	Strong
	VP-16	1.0		0.78	0.14	Strong
		10.0		0.85	0.06	Very strong
		0.1		0.73	0.18	Strong
	Doxorubicin	1.0		0.81	0.14	Strong
		2.0		0.83	0.16	Strong

Values under *Effect* (fifth column) denote the proportion of cells that were killed by the combination treatment.

### Analysis of the synergistic killing effect of AEW541 and gemcitabine

**Figure 4:**
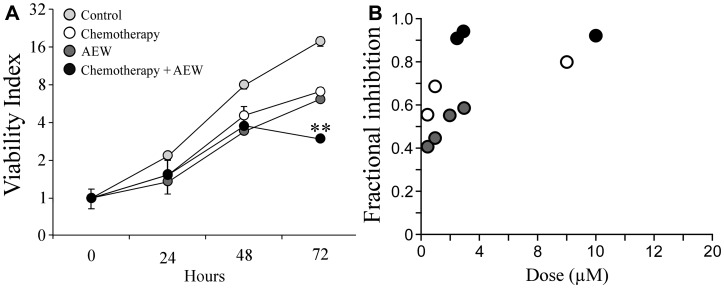
Synergistic effect of AEW541 and gemcitabine co-treatment on cell viability. (**A**) MCF7 cells were seeded in 96-well plates at a density of 3 × 10^3^ cells per well. After 24 h, cells were treated with 1 μM AEW541 and 5 nM gemcitabine, alone or in combination. After 72 h, cell viability was measured using an SRB assay. Each point represents the mean ± SEM of three independent experiments. Statistical analysis was performed by using unpaired Student’s *t*-test (^**^ *p <* 0.01 co-treatment *versus* single-agent treatment; single-agent treatment *versus* control). The CI values were determined by the *Compusyne* software. The y-axis is at a logarithmic scale. (**B**) Fractional inhibition is calculated as the *minimum inhibitory concentration* (MIC) of drug in combination divided by the MIC of drug acting alone.

The effect of combined treatment of AEW541 with gemcitabine on MCF7 cell viability was next examined by sulforhodamine B (SRB) assays using a lower dose of the inhibitor (1 μM instead of 2 μM). The rationale for this analysis was the fact that AEW541 exhibits a dose-dependent toxicity. Even at this lower dose, AEW541 monotherapy significantly reduced cell viability in comparison to control cells at 72 h. Combined treatment with gemcitabine led to a significantly enhanced killing effect (compared to each one of the single agent treatments) (Figure 4A). The synergistic effect was even more pronounced when examining the fractional inhibition of the treatments (Figure 4B and Table 2). Fractional inhibition is an expression of the synergy between drugs and is calculated as the *minimum inhibitory concentration* (MIC) of drug in combination divided by the MIC of drug acting alone.

**Table 2:** CI values for the synergistic effect of AEW541 and gemcitabine

Cells	Drug	Drug Dose (uM × 10)	Dose AEW (uM)	Effect	CI	Synergism
MCF7	Gemcitabine	0.50	1.00	0.92	0.01	Very strong
		1.00	1.00	0.95	0.00	Very strong
		10.00	1.00	0.93	0.04	Very strong

### Effect of combined AEW541 and gemcitabine treatment on the cell cycle

**Figure 5:**
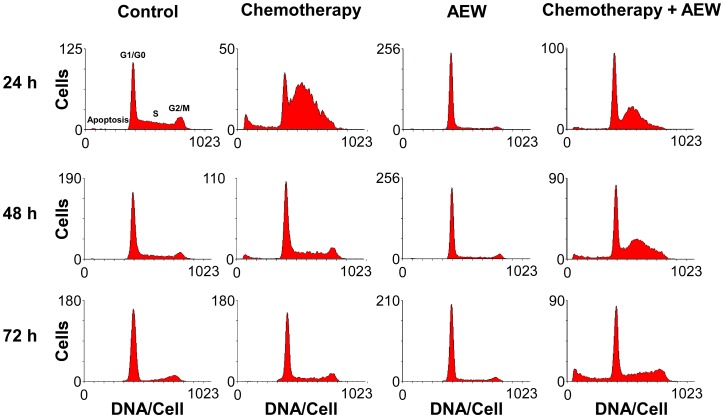
Effect of combined treatment on MCF7 cell cycle. MCF7 cells were seeded in 10-cm plates at a density of 2 × 10^6^ cells per plate for 24 h. The cells were then treated with AEW541 (2 μM), or gemcitabine (5 nM), or both for 24, 48 and 72 h. At the end of the incubation period, cells were collected by trypsinization into their own medium to prevent loss of dead cells. Cells were fixed with 70% ice-cold ethanol, stained with propidium iodide and used for cell cycle and apoptosis analyses performed by FACS Calibur system. Graphs present the number of cells *versus* the amount of DNA, as indicated by the intensity of a fluorescence signal, upon each treatment.

To identify possible mechanisms responsible for the synergic effect, the cell cycle distribution of MCF7 cells after treatment with AEW541 and gemcitabine, alone or in combination, was investigated. As shown in Figure 5, AEW541 led to G1 arrest after 24 h whereas gemcitabine caused S arrest. In both cases, the cells survived following 72 h of monotherapy, but the combined treatment led to apoptosis after 72 h. These results suggest that the synergistic affect with ensuing apoptosis stems, at least in part, from the targeting of different phases of the cell cycle by the two agents.

### Proteomics analyses

To further investigate the synergistic effect following co-treatment of AEW541 and gemcitabine, we focused on the identification and quantitation of the proteins associated with pro-death and pro-survival signaling pathways. For this purpose, MCF7 cells were treated with either agent as monotherapy or in combination, and the global expression alterations *in vitro* were evaluated at two different time points (24 and 48 h), as described under *Materials and Methods*. Untreated MCF7 cells were used as controls. This analysis detected 3530 proteins, and following implementation of predefined criteria (*p* value < 0.05 and fold-change difference ≥ 1.5) a total of 312 proteins were selected.

### Effect of single agent versus combined treatment on protein expression

**Figure 6:**
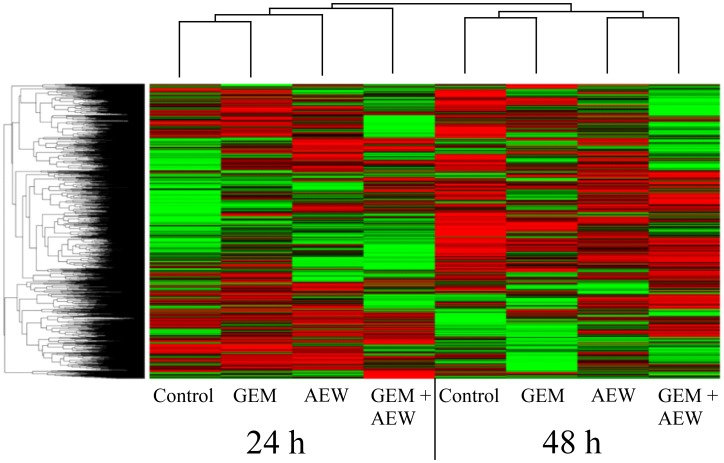
Classification of protein expression by clusters. Heatmap and dendrograms were constructed by using MATLAB’s clustergram function. Rows and columns represent protein types and samples at 24 and 48 h. The row tree represents proteins and the column tree represents the treatments. The colors in the heat table represent the intensities of the underlying protein expression. The data has been normalized across all samples for each protein, so that the mean is 0 and the standard deviation is 1. The heatmap color key is: red - value higher than average, green - value lower than average, black - value equal to the average value and gray - cells in the input table that did not have values in the first place. GEM, gemcitabine; AEW, AEW541; GEM+AEW, combined treatment.

Heatmaps were created to visualize the overall expression patterns of the proteins (Figure 6). Clear differences were observed between differentially expressed proteins at 24 and 48 h, indicating that time was a key parameter in this experiment. In addition, AEW541 treatment was clustered with AEW541 + gemcitabine treatment at both time points, suggesting a synergistic effect. Finally, analyses established that the co-treatment involved two sets of proteins related to the apoptotic process that were either up- or down-regulated and that are most probably involved in promoting the synergistic effect.

Volcano plot analysis of expression data was used to generate six different plots for the different treatments at the various times. The results of these analyses are presented under *Supporting information* (Supplementary Figure 2 and Supplementary Table 1). Venn diagrams of differentially expressed proteins and a graphical representation of up- and down-regulated proteins are presented in Supplementary Figure 3. These analyses identified a more pronounced synergistic effect at 48 than at 24 h. Furthermore, the existence of a marked synergism is supported by the finding that more than twice as many differentially expressed proteins were obtained following the combination treatment than after each individual treatment.

K-means clustering was employed to assemble groups of proteins with similar profiles. Out of five clusters identified at 48 h, two were altered following combination treatment. Specifically, cluster 1 includes 59 up-regulated proteins and cluster 3 includes 80 down-regulated proteins. K-means analysis is presented under *Supporting information* (Supplementary Figure 4). The proteins included in clusters 1 and 3 are listed in Supplementary Table 2. A number of Gene Ontology enrichment bioinformatics tools were employed in order to identify specific proteins associated with the synergistic killing effect (see *Supporting information*, Supplementary Tables 3 and 4).

### Clinical significance of IGF1R levels

**Figure 7:**
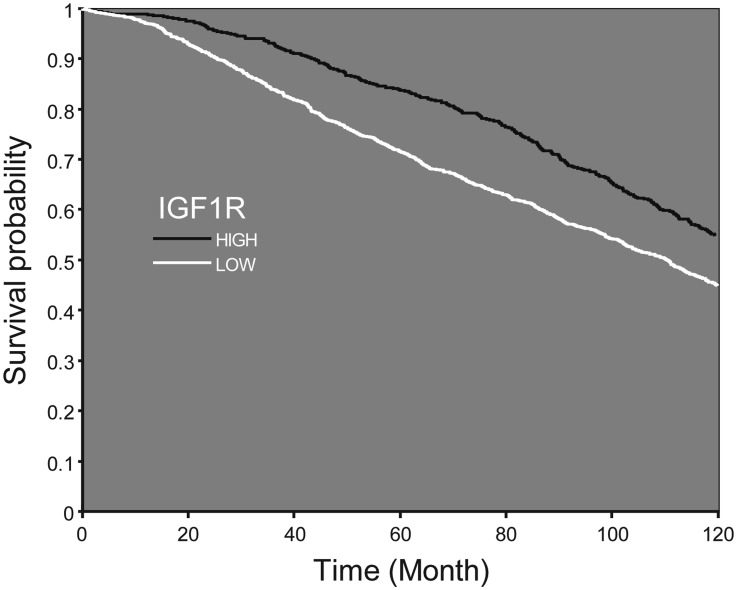
Survival probability according to IGF1R mRNA expression. Kaplan–Meier analysis of survival probability of breast cancer patients expressing low or high IGF1R mRNA values. Patients expressing high levels of IGF1R demonstrate a shift of the curve to the right, which accounts for higher survival probability from initial diagnosis till 10 years later.

A Kaplan-Meier analysis was performed on a database of patients with breast cancer (www.cbioportal.org; *n =* 1904), expressing low (LOW1) or high (HIGH1) levels of IGF1R mRNA. Survival curves were constructed to compare between high *versus* low IGF1R mRNA. As shown in Figure 7, high IGF1R was significantly associated with greater survival probability (*p <* 0.001).

**Figure 8:**
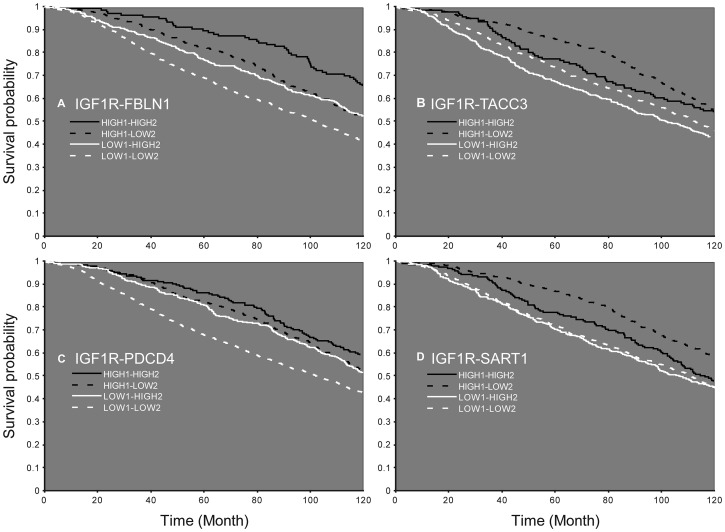
Survival probability according to the expression of IGF1R mRNA and an additional gene. Patients expressing low (LOW1) or high (HIGH1) levels of IGF1R and FBLN1 (**A**), TACC3 (**B**), PDCD4 (**C**), or SART1 (**D**). Patients with high levels of the second gene are denoted HIGH2 and patients with low levels of the second gene are denoted LOW2.

Finally, analyses identified 164 mRNAs whose expression significantly differed between groups (low or high IGF1R mRNA), including 24 cases with high-confidence level (Supplementary Table 5). Based on this data, survival curves were constructed for the combination of IGF1R and each of the 164 mRNAs. This analysis identified ten genes that differed significantly (*p <* 0.05) in all four groups (ADSS, CC2D1A, FBLN1, HMGN2, LARS, PDCD4, RAB25, SART1, TACC3, and USP15) (Table 3). As mentioned above, patients with high IGF1R levels had a greater survival probability than those with low levels, while high levels of FBLN1 further increased survival. On the other hand, high levels of TACC3 reduced survival chances (Figure 8A, 8B). In addition, high levels of PDCD4 were associated with increased survival probability in low IGF1R patients, while high levels of SART1 were associated with decreased survival probability in high IGF1R patients (Figure 8C, 8D).

**Table 3:** List of ten genes with significant differences in all four groups (*p <* 0.05)

mRNA	HIGH1-HIGH2 (*n*)	HIGH1-LOW2 (*n*)	LOW1-HIGH2 (*n*)	LOW1-LOW2 (*n*)	mRNA2 *p*-value	Confidence level
**FBLN1**	132	440	440	892	8.68619E-07	High
**ADSS**	128	444	444	888	1.7E-05	Low
**PDCD4**	205	367	367	965	7.37794E-06	Low
**CC2D1A**	212	360	360	972	1.13348E-05	Low
**TACC3**	141	431	431	901	1.396E-05	Low
**USP15**	188	384	384	948	1.5364E-05	Low
**HMGN2**	204	368	368	964	1.61573E-05	Low
**LARS**	195	377	377	955	1.64125E-05	Low
**ADSS**	128	444	444	888	1.69623E-05	Low
**RAB25**	188	384	384	948	1.76618E-05	Low

The values in columns 2–5 denote the number of patients in each group.

## DISCUSSION

IGF1 has been recognized as a major regulator of mammary epithelial cell and breast cancer growth [28]. The IGF1R, which mediates the pro-survival actions of IGF1, constitutes a key player in breast carcinogenesis [29]. The central role of IGF1R in malignant transformation has been illustrated by seminal studies showing that overexpression of the receptor in fibroblasts resulted in a ligand-dependent, highly transformed phenotype that included the formation of tumors in nude mice [30]. Furthermore, fibroblasts cell lines established from mouse embryos in which the IGF1R was disrupted by homologous recombination did not undergo transformation when exposed to different oncogenes [31]. These early studies positioned the IGF1R at a critical node in the area of oncology and provided a conceptual rationale for targeting studies.

As mentioned above, most Phase III clinical trials conducted over the past 15 years using either IGF1R-directed antibodies or selective IGF1R inhibitors in unselected patients led to disappointing results [17, 19, 32]. As a result of these negative outcomes an urgent need to identify predictive biomarkers that may identify potential responders emerged. Furthermore, it became increasingly evident that it might be necessary to assess the differential effects of monotherapy as compared to combination therapies [33]. In addition, the administration of combination therapy at specified time intervals between drugs merits further investigation. The present study provides evidence that co-treatment of IGF1R inhibitor along with chemotherapeutic drugs improves the treatment efficiency in breast cancer cells expressing high levels of IGF1R. Specifically, data indicate that the degree of synergy achieved by AEW541 and chemotherapy, as expressed in combination index (CI) values, was very strong. Moreover, the pyrimidine analogue gemcitabine produced the strongest synergistic effect (Tables 1 and 2). Our bioinformatic analyses shed light on some of the biological pathways that might be responsible for this synergy, including identification of two candidate protein lists that may play a role in the killing effect and apoptosis following the combined treatment.

Cell cycle analyses suggest that the synergism was derived, at least in part, from AEW541-induced G1 arrest and gemcitabine-induced S arrest. Gemcitabine is a pyrimidine antimetabolite that inhibits DNA synthesis [34], and its combination with paclitaxel is a preferred chemotherapeutic regimen for patients with metastatic breast cancer [35]. Preoperative combination of gemcitabine, carboplatin and iniparib is active in the treatment of early-stage triple-negative and BRCA1/2 mutation-associated breast cancer [36]. Despite numerous attempts, most gemcitabine combinations with molecularly targeted therapies have failed to demonstrate a significant improvement in overall survival, with the exception of gemcitabine + erlotinib (an EGFR tyrosine kinase inhibitor), which has demonstrated a statistically significant but clinically modest benefit [37]. Here, the synergistic killing effect may be due to violation of the balance between cell survival and cell death induced by DNA damage (gemcitabine) along with inhibition of the survival machinery of the cells (AEW541). In accordance with our results, *in vitro* studies in MCF7 cells reported effectiveness when using a selective IGF1R tyrosine kinase inhibitor (PQIP) in combination with gemcitabine [38]. Finally, enhancement of the response to other IGF1R monoclonal antibodies by gemcitabine has been demonstrated in several cancers, including breast tumors [39, 40].

In terms of the global expression alterations following combined therapy, length of treatment emerged as a key factor as the expression profiles following all three treatments (AEW541, gemcitabine or combination therapy) are markedly different, with little or no overlap between proteins expressed at both time points. Moreover, while there was no overlap between proteins regulated by AEW541 and gemcitabine at 24 h, several overlaps emerged at the 48 h time point. One possible explanation for this finding is the fact that the treatment induced an acute effect that was then counterbalanced by a compensation process. For example, it has been shown that in epithelial tissue, apoptosis is often compensated by increased proliferation to maintain the tissue structure [41]. In the present study, the combined treatment induced up-regulation of proteins associated with mitosis at 48 h and down-regulation of proteins associated with the 19S regulatory complex of the 26S proteasome, which catalyzes protein degradation [42]. The proteasome’s 19S regulatory cap binds the polyubiquitin chain, denatures the protein, and feeds the protein into the proteasome’s proteolytic core [43]. As such, down-regulation of these proteins may reduce protein degradation.

Network analyses identified two subnetworks of both up- and down-regulated proteins related to amyloid precursor protein (APP) and the epidermal growth factor receptor (EGFR). APP and its processing enzymes were shown to be linked with breast cancer via Akt phosphorylation [44]. Thus, APP is involved in the proliferation of cancer cells as well as in their adherence and movement and its exaggerated synthesis is considered a potential prognostic factor in ER-positive breast cancer patients [45]. Studies suggested that IGF1R and EGFR combinatorial therapy might constitute a promising approach for cancer. In view of the fact that there is cross-talk between both growth factors, combinations have the potential to be more effective than targeting each factor separately [39]. Given that overexpression of EGFR and ERBB2 by breast tumors predicts a poor prognosis, it might be possible in the future to predict response to IGF1R-directed therapy based on ERBB2/EGFR status [46].

Growing clinical evidence suggests potential correlations between biomarkers related to the IGF1R pathway and clinical benefits from IGF1R-targeted therapies [32, 33]. Thus, IGF1R levels were differentially expressed with variable prognostic impact among breast cancer subtypes [11, 47]. In addition, high IGF1R expression and elevated circulating IGF1 levels were correlated with improved response to IGF1R-targeted therapies in clinical trials for non-breast cancers [48, 49]. Moreover, studies in sarcoma and neuroblastoma cell lines showed that sensitivity toward a specific tyrosine kinase inhibitor (BMS-536924) was associated with high basal IGF1R expression [39]. IGF1R expression in circulating tumor cells was associated with response to IGF1R targeting in patients with advanced prostate cancer [50]. Of interest, Obr et al [51] recently identified an association between low IGF1R expression and reduced overall patient survival. The authors provide evidence that inhibiting IGF1R in either mouse or human tumor epithelial cells increased reactive oxygen species production and activation of the endoplasmic reticulum stress response. Hence, reduction of IGF1R may lead to enhanced cellular stress and cytokine production, with ensuing promotion of an aggressive tumor microenvironment. Finally, increased nuclear localization of IGF1R was associated with better overall survival for patients treated with IGF1R therapy [52]. In this context, we have recently provided evidence that the capacity of a specific IGF1R antibody to block IGF1-mediated IGF1R activation was impaired in mutant BRCA1-expressing breast cancer cells [25]. Hence, the mutational status of BRCA1 must be taken into account when selecting patients for IGF1R targeting protocols.

The use of a breast cancer clinical database allowed us to identify several genes whose co-expression with IGF1R also affected life expectancy. It is of interest to note that all ten genes identified (Table 3), as well as IGF1R, are downstream targets of the early growth response-1 (EGR1) gene [53]. EGR1 is a stress response transcription factor with multiple tumor suppressor roles in breast tissue. The expression of EGR1 is often lost in breast cancers [54]. EGR1 directly binds to the human IGF1R gene promoter, regulates its expression, activates the ERK and AKT pathways and promotes cancer cell growth. EGR1 may also be a target for directed therapy [55]. Of interest is the fact that while IGF1 activates the transduction network mediated by IGF1R leading to the up-regulation of EGR1 [56], impaired IGF1 signaling (caused by IGF1R mutation) resulted in a reduced induction of EGR1 [57]. Furthermore, IGFBP-3 suppresses transcription of EGR1 through inhibition of IGF1-dependent ERK and AKT activation [58]. Taken together, these results are consistent with a complex, bi-directional feed-back loops involving the EGR1 and IGF1 signaling pathways.

In summary, our study provides evidence that co-treatment of IGF1R inhibitor along with chemotherapeutic drugs greatly improves the treatment efficiency in breast cancer cells expressing high level of IGF1R. Data suggest that high IGF1R baseline expression may serve as a predictive biomarker for IGF1R targeted therapy. While many clinical trials conducted in recent years have shown that IGF1R-directed monotherapy is a poor therapeutic approach, we show that combining this therapy with chemotherapy leads to a potent synergistic effect. Bioinformatics analyses shed light on some of the biological pathways that might be responsible for this synergy. Finally, corroborating the results in the clinical setting may be a step towards personalized oncological treatments.

## MATERIALS AND METHODS

### Cell lines

The MCF7 cell line (ER+, PR+) is an aggressive adenocarcinoma line derived from a metastatic site [59]. Cells were obtained from the American Type Culture Collection (Manassas, VA, USA) and maintained in high glucose DMEM supplemented with 10% fetal bovine serum (FBS), 2 mM l-glutamine and antibiotics. Cells were propagated in a 37°C humidified incubator with 5% CO_2_.

### IGF1R inhibitors

MK-0646 (Dalotuzumab; Merck, Sharp and Dohme Ltd., Whitehouse Station, NJ, USA) and A12 (Cixutumumab; ImClone Systems, New York, NY, USA) are humanized IgG1 monoclonal antibodies antagonist to the human IGF1R [60, 61]. The antibodies were diluted in 20 mM histidine and 150 μM NaCl and used at a concentration of 10 mg/ml. AEW541 (Novartis Pharma, Basel, Switzerland) and AG1024 (Sigma-Aldrich Ltd, St. Louis, MO, USA) are selective IGF1R tyrosine kinase inhibitors. AEW541 and AG1024 were kept as a stock solution (10 mM) in DMSO and stored at –20° C. AEW541 is a reversible, ATP-competitive phosphorylation inhibitor that exhibits high selectivity towards IGF1R over insulin receptor [62]. AG1024 belongs to the tyrphostin family of tyrosine kinase inhibitors [63].

### Anti-cancer drugs

Doxorubicin is an anthracycline antibiotic drug widely used in clinical practice to treat several types of cancers [64]. Treatment with doxorubicin is a present standard of care for patients with metastatic soft-tissue sarcoma. Gemcitabine is a pyrimidine analogue and a ribonucleotide reductase inhibitor that has a broad spectrum of anti-tumor activity, including first-line treatment option for recurrent or metastatic nasopharyngeal carcinoma [65]. Taxol is an effective anticancer agent that stabilizes microtubules and prevents them from depolymerizing. It is used to treat various cancers including ovarian, breast, lung, and head and neck [66]. Irinotecan is a semisynthetic camptothecin product that inhibits DNA topoisomerase I [67]. Cisplatin is an alkylating-like drug that crosslinks with the purine bases, resulting in distortion of the DNA structure. This interferes with DNA repair mechanisms, causing DNA damage and, subsequently, apoptosis in cancer cells. Cisplatin is a potent chemotherapeutic agent used in standard medulloblastoma protocols [68]. VP-16 is is a topoisomerase II inhibitor and DNA synthesis inhibitor that is used in the treatment of many cancer types [69].

### Western immunoblots

Cells were seeded at a concentration of 3 × 10^3^ cells per well in 96-well plates or 1 × 10^6^ cells per plate in 10-cm plates and exposed to the indicated treatments after 24 h. Cells were collected after an additional 24–72 h by scraping, washed with ice-cold phosphate-buffered saline (PBS), and lysed with RIPA buffer (150 mM NaCl, 1% NP-40, 0.5% deoxycholic acid, 0.1% SDS, 0.5 M Tris pH 8), supplemented with complete mini-protease inhibitor cocktail (Roche Diagnostics GmbH, Mannheim, Germany). Protein concentration was determined with the Pierce BCA protein assay kit (Thermo Scientific, Rockford, IL, USA). Samples (50 μg) were resolved on 6% and 10% SDS-PAGE, transferred to Protran BA-83 0.2 μm nitrocellulose membranes (Whatman, Piscataway, NJ, USA), blocked with 5% skim milk and immunoblotted with antibodies against phospho-IGF1R (Cat. 3024, directed against Tyr1135/1136), total-IGF1R β-subunit (Cat. 3027), phospho-AKT (pAKT; Cat. 9271, against Ser473), total AKT (Cat. 9272), and phospho-ERK1/2 (pERK; Cat. 9106, against Thr202/Tyr204). Antibodies were obtained from Cell Signaling Technology (Beverly, MA, USA). Antibodies against total ERK1/2 (Cat. K23), actin (I-19; sc-1616), and Cbl (C-15; sc-170) were from Santa Cruz Biotechnology (Dallas, TX, USA). Membranes were washed, incubated with the corresponding horseradish peroxidase-conjugated secondary antibody, probed with an enhanced chemiluminescence detection reagent and exposed to Fuji Super RX film (Tokyo, Japan). The expression of β-actin, tubulin or Cbl was measured as a loading control.

### Proliferation assays

Cells were plated in triplicate in 96-well plates (3 × 10^3^ cells/well) and allowed to attach overnight. The medium was replaced with fresh treatment-containing medium and the cells were propagated for an additional 72 h. Cell viability was determined using an XTT cell proliferation kit (Biological Industries) by replacing the medium with fresh medium containing charcoal-stripped FBS (in order to prevent interference of treatment color with the XTT signal), and the addition of XTT for 2–3 h according to the manufacturer’s instructions. The resulting signal was measured by a Power Wave × 340-I ELISA reader (Biotek Instruments, Winooski, VT, USA) in at least three independent assays.

### Sulforhodamine B (SRB) viability assays

For SRB viability assays, cells were fixed with 10% trichloroacetic acid for 1 h, washed extensively with dd water, dried and stained with 0.057% sulforhodamine B (w/v in 1% acetic acid) for 1 h. Following staining, the cells were washed with 1% acetic acid, after which 200 ml of 10 mM Tris was added to each well to solubilize SRB. The absorbance was measured at 570 nm using an ELISA reader. The viability index for each individual treatment was calculated by dividing the values obtained for the treated cells by the values of the untreated cells. Experiments were repeated three times.

### Cell cycle analysis

Cells were seeded onto 6-well plates (0.5 × 10^6^ cells/well) for 24 h. Cells were then serum starved for an additional 24 h and incubated in the presence or absence of IGF1 with or without MK-0646 for 24 h. After incubation, cells were washed with PBS, trypsinized, centrifuged, resuspended in citrate buffer, and stored at –80°C prior to analysis. The cells were thawed and permeabilized before adding propidium iodide. The dye intercalates into cellular DNA and the intensity of the signal is directly proportional to DNA content. Stained cells were analyzed using a FacsCalibur system (Cytek Development Inc., Fremont, CA, USA). The events were evaluated for each sample and the cell cycle distribution was analyzed using the Cell Quest software (Becton Dickinson). The results are presented as the number of cells *versus* the amount of DNA, as indicated by the intensity of a fluorescence signal.

### CompuSyn software for CI values

The *CompuSyn* software (CompuSyn Inc, Paramus, NJ, USA) was used for automated quantitative simulation of synergism or antagonism in drug combination studies. *CompuSyn* calculates the dose-effect curves and the combination index (CI) plot for effect-oriented determination of synergism or antagonism at different effect levels [70].

### Proteomics analyses

Samples of proteins expressed following monotherapy or combination therapy were collected at two time points (24 and 48 h). The experiments were performed in triplicate using three different experiment batches. Samples were lysed and global quantification of total proteome was conducted by liquid chromatography-mass spectrometric (LC-MS) analysis [71]. The analyses were conducted at the National Center for Personalized Medicine, Weizmann Institute of Science, Rehovot, Israel.

### Data analysis

For protein expression, raw data was imported into the Expressionist® software (Genedata) [72]. The software was used for retention time alignment and peak detection of precursor peptides. A master peak list was generated from all MS/MS events and sent for database searching using Mascot v2.5 (Matrix Sciences). Data was searched against the human protein database as downloaded from UniprotKB (http://www.uniprot.org/), appended with 125 common laboratory contaminant proteins. Fixed modification was set to carbamidomethylation of cysteines and variable modifications were set to oxidation of methionines and deamidation of N or Q. Search results were then filtered using the PeptideProphet algorithm to achieve maximum false discovery rate (FDR) of 1% at the protein level [73]. Peptide identifications were imported back to Expressionist to annotate identified peaks. Quantification of proteins from the peptide data was performed using an in-house script [73]. Data was normalized base on the total ion current. Protein abundance was obtained by summing the three most intense, unique peptides per protein. A Student’s t-Test, after logarithmic transformation, was used to identify significant differences across the biological replica. Fold-changes were calculated based on the ratio of arithmetic means of the case versus control samples.

### Bioinformatics analyses

The bioinformatics tools employed are described under *Supporting information*.

### Kaplan–Meier analysis

The Kaplan-Meier analysis was performed using the cBioPortal for Cancer Genomics database (www.cbioportal.org). The total database includes 2509 patients, 74.3% of which were diagnosed with invasive ductal breast carcinoma, 10.7% with mixed ductal and lobular breast carcinoma, and 7.7% with invasive lobular breast carcinoma. The patients analyzed here (*n =* 1904) were 25 to 95 years old (average = 61.1 ± 12.9 y), with an overall survival of 125.2 ± 76.1 months since first diagnosis, and with Nottingham prognostic index of 4.0 ± 1.1. Data was analyzed according to IGF1R expression, such that 70% patients (*n =* 1332) were characterized as LOW1 and 30% (*n =* 572) were characterized as HIGH1. The average expression of IGF1R mRNA was calculated for each group and a p-value was obtained for the difference in IGF1R expression (p-IGF1R). The groups were then compared for their expression of other genes derived from the database, and significant different expression between the groups of a different mRNA was retrieved according to the following criteria: low-confidence - *p*-value < 0.05; high-confidence - *p*-value < 0.05 and *p*-value < p-IGF1R. For each mRNA identified, similar allocation of patients into subgroups (i. e., lower 70% and upper 30%) was conducted. Survival curves were constructed for the main mRNA being examined (IGF1R; LOW1 vs HIGH1), and for the combination of IGF1R and each of the 164 mRNAs (LOW2 vs HIGH2) identified with confidence using clinical data from the database.

### Statistical analyses

The statistical significance of differences was assessed by Student’s *t*-test (two samples, equal variance). Results are presented as mean ± SEM of three independent experiments, performed in triplicate dishes. A *p*-value of 0.05 was considered statistically significant.

## SUPPLEMENTARY MATERIALS






