# Supplementary material for: Proteomic analysis of combined IGF1 receptor targeted therapy and chemotherapy identifies signatures associated with survival in breast cancer patients
Source: Oncotarget. 2020 Apr 28;11(17):1515–30. doi: 10.18632/oncotarget.27566 (PMC7197451; doi:10.18632/oncotarget.27566)
Supplement: Supplementary file 1 [file oncotarget-11-1515-s001.pdf]

Proteomic analysis of combined IGF1 receptor targeted therapy and chemotherapy identifies signatures associated with survival in breast cancer patients

SUPPLEMENTARY MATERIALS

SUPPORTING MATERIALS AND METHODS

Bioinformatics tools

Heatmaps

Heatmaps and dendrograms were constructed by using the MATLAB's clustergram function. Each color represents the normalized data of protein expression. Rows and columns represent protein types and samples at two different time points, respectively. The row tree represents proteins and the column tree represents the treatments. The colors in the heat table represent the intensities of the underlying protein expression. The input received by the clustergram function is a table with numerical values (intensity values). The data has been normalized across all samples for each protein, so that the mean is 0 and the standard deviation is 1. The heatmap colors indicate values higher than average, lower than average, equal to the average, and cells in the input table that did not have values in the first place.

Venn diagrams

Venn diagrams (http://bioinfogp.cnb.csic.es/tools/venny/) were used to identify overlapping protein signature responses to the different treatments. A threshold of $p < 0.05$ and fold-change difference ≥ 1.5 was set as a significant differential expression.

Volcano plots of expression-proteomic data

A volcano plot is a type of scatter plot that is used to quickly identify changes in large data sets composed of replicate data. It plots significance versus fold-change on the y and x axes, respectively. A volcano plot combines a measure of statistical significance from a statistical test (*p* value from an ANOVA model) with the magnitude of the change enabling quick visual identification of proteins with large magnitude changes and statistically significant. Each protein is represented as a dot in these plots.

K-means clustering

K-means clustering is used on unlabeled data or data without defined categories and groups. K-means clustering aims to divide n objects into k clusters in which each object belongs to the cluster with the nearest mean, serving as a prototype of the cluster. Data points are clustered based on feature similarity. Rather than defining groups before looking at the data, clustering allows to find and analyze the groups that have formed organically. Here, K-means clustering was used to group proteins with similar profiles. The rows represent proteins and the columns represent the treatment in three biological repeats. Up-regulated proteins were marked in red and down-regulated proteins were marked in blue.

GeneAnalytics

GeneAnalytics (geneanalytics.genecards.org) provides expression and function-based enrichment analysis for gene sets. GeneAnalytics is powered by GeneCards (human gene database), LifeMap Discovery (embryonic development and stem cells database), MalaCards (human diseases database) and PathCards (biological pathways database). These databases contain annotated gene lists for tissues and cells, diseases, pathways, compounds and gene ontology terms. GeneAnalytics compares specific gene sets to these compendia in search of the best matches. The output contains the best matched gene lists, scored and subdivided into their biological categories such as diseases or pathways. This tool was used to understand how the different treatments affected MCF7 cells at the proteomic level.

SUPPORTING DATA

Volcano plot analysis of expression (proteomic) data

Six different plots were obtained for the different treatments at the different times (Supplementary Figure 2). After 24 h, treatments with AEW541, GEM or AEW541 + GEM caused significant changes in the expression of 115, 55, and 159 proteins, respectively; and after 48 h caused significant changes in the expression of 91, 93 and 220 proteins, respectively (for details see Supplementary Table 1S).

Venn diagram of the expression (proteomic) data

Venn diagram analysis generated six lists of proteins at each time point. At the 24 h time point, common proteins were only observed for AEW and the combined treatment,

but at the 48 h time point, common proteins were observed for all combinations (Supplementary Figure 3A, 3B). These results can be explained by the different proteins that were expressed following the different treatments at the two time points.

Candidate putative up- and down-regulated proteins

Next, we evaluated the proportion of proteins that were up- or down-regulated after 24 and 48 h of treatment (Supplementary Figure 3C). As can be seen, the various treatments led to different patterns of up- and down-regulated expression.

K-means clustering analysis

To cluster the proteins that were up- or down-regulated we used K-means clustering and were able to identify five clusters of proteins at the 48 h time-point (Supplementary Figure 4). Of interest, two clusters of proteins that were altered following treatment with AEW541+ GEM were identified. Cluster 1 (red box) is a group of up-regulated proteins and cluster 3 (blue box) is a group of down-regulated proteins (The protein lists are displayed in Supplementary Table 2).

Analysis of up-regulated proteins

Gene Ontology analyses identified a number of categories for biological processes and molecular functions that are strongly associated with the up-regulated proteins (Supplementary Table 3). Among other proteins, our analyses identified the KIFC1, KIF11 and TPX2 proteins. KIFC1 and its opposing motor KIF11 have an important role in spindle assembly and the bi-polar arrangement of mitotic microtubules [1]. Interestingly, both KIFC1 and KIF11 mRNAs were up-regulated in aggressive tumors and were associated with shorter progression-free survival in meningioma tissues [2].

Analysis of down-regulated proteins

Gene Ontology analyses identified a number of proteins in various categories that are down- regulated upon combined treatment. For example, PSMC2, PSMC1, PSMD2, and PSMC4 are regulatory subunits of the 26S proteasomes and its 19S regulatory complex, which is part of a cellular protein degradation pathway that plays a vital role in many cellular functions [3].

REFERENCES

1. Singh SA, Winter D, Kirchner M, Chauhan R, Ahmed S, Ozlu N, Tzur A, Steen JA, Steen H. Co-regulation proteomics reveals substrates and mechanisms of APC/C-dependent degradation. EMBO J. 2014; 33:385–99. https://doi.org/10.1002/embj.201385876. [PubMed]
2. Jungwirth G, Yu T, Moustafa M, Rapp C, Warta R, Jungk C, Sahm F, Dettling S, Zweckberger K, Lamszus K, Senft C, Loehr M, Keßler AF, et al. Identification of KIF11 As a Novel Target in Meningioma. Cancers (Basel). 2019; 11. https://doi.org/10.3390/cancers11040545. [PubMed]
3. Soave CL, Guerin T, Liu J, Dou QP. Targeting the ubiquitin-proteasome system for cancer treatment: discovering novel inhibitors from nature and drug repurposing. Cancer Metastasis Rev 2017, 36:717–36. http://doi.org/10.1007/s10555-017-9705-x. [PubMed]

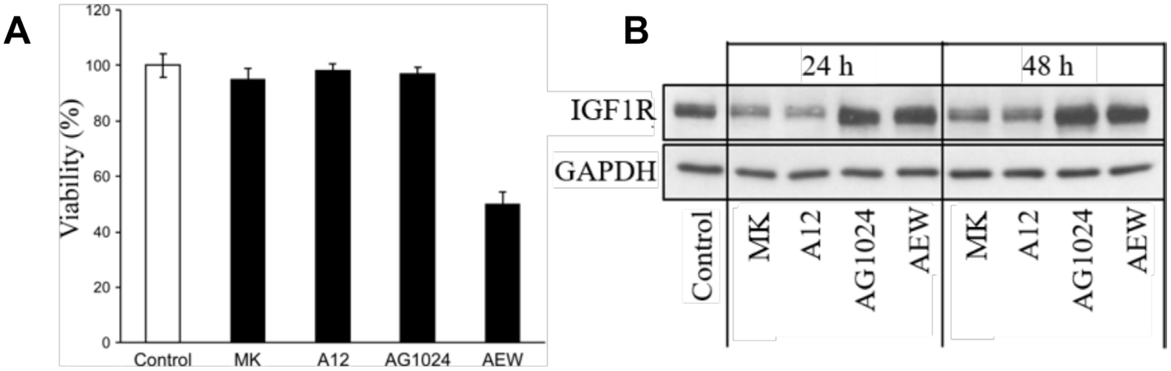

Supplementary Figure 1: Effect of IGF1R inhibitors on cell viability and IGF1R protein expression. (**A**) MCF7 cells were seeded in 96 well-plates at a density of 3×10^3 cells per well for 18 h. Cells were then treated with each of four IGF1R inhibitors [MK-0646 (10 µg/ml), A12 (10 µg/ml), AG1024 (10 µM) and AEW541 (3 µM)] separately. Cell viability was measured after 48 h using an XTT assay. (**B**) Cells were seeded in 10-cm plates at a density of 1×10^6 cells per plate for 18 h. Cells were then treated with each of the IGF1R inhibitors for 24 and 48 hr. At the end of the incubation period, cells were lysed and IGF1R levels were measured by Western blot analysis. Equal loading was confirmed by GAPDH loading.

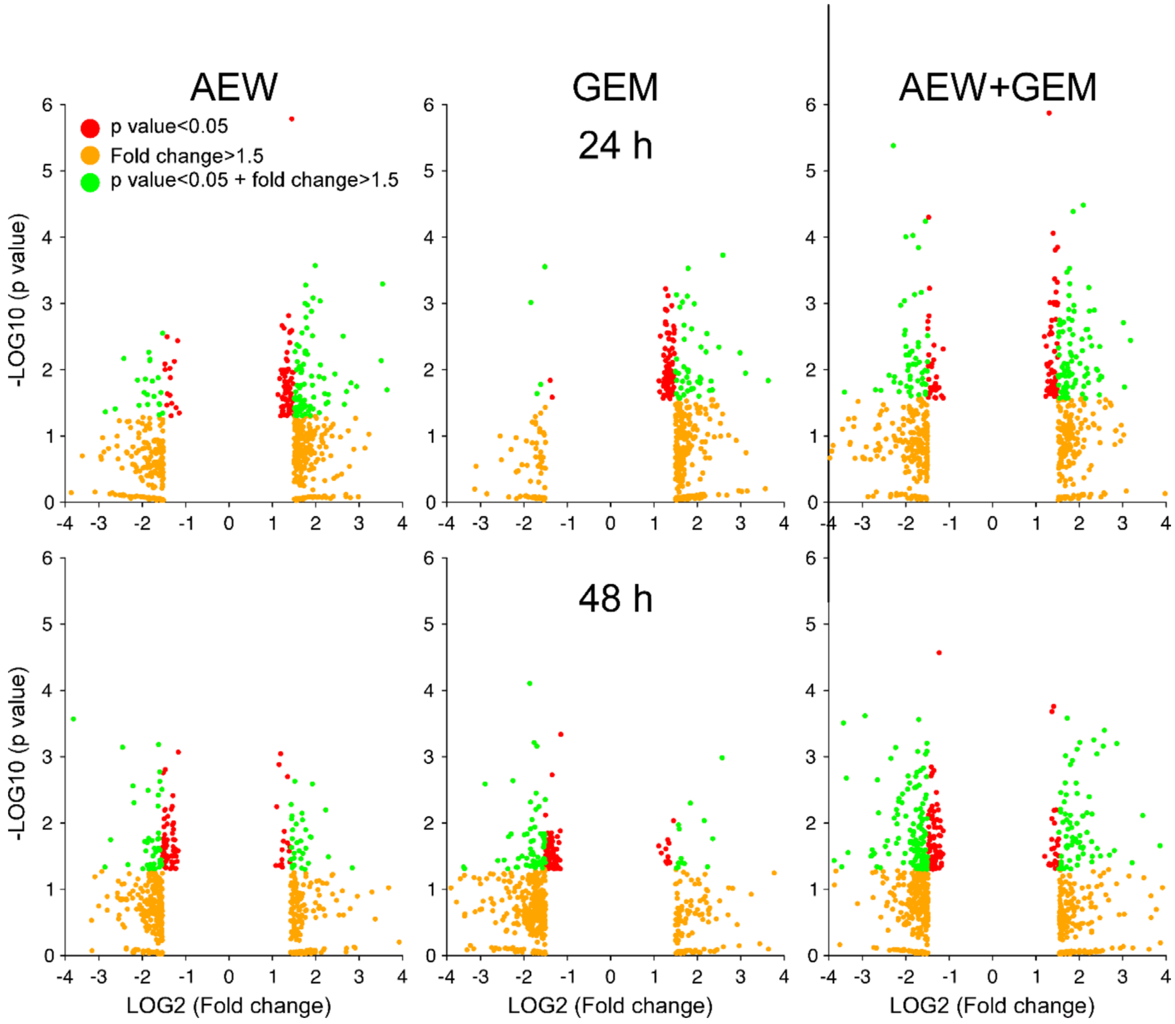

Supplementary Figure 2: Volcano plot of expression data. Volcano plot analysis was used to generate plots for the different treatments at both time points. After 24 h, treatments with AEW541, GEM or combined therapy caused significant changes in the expression of 115, 55 and 159 proteins, respectively. After 48 h, the various treatments caused changes in the expression of 91, 93 and 220 proteins, respectively. The fold change (x axis) is plotted against statistical significance (y axis) for each protein in each treatment. The list of proteins depicted here is presented in Supplementary Table 1.

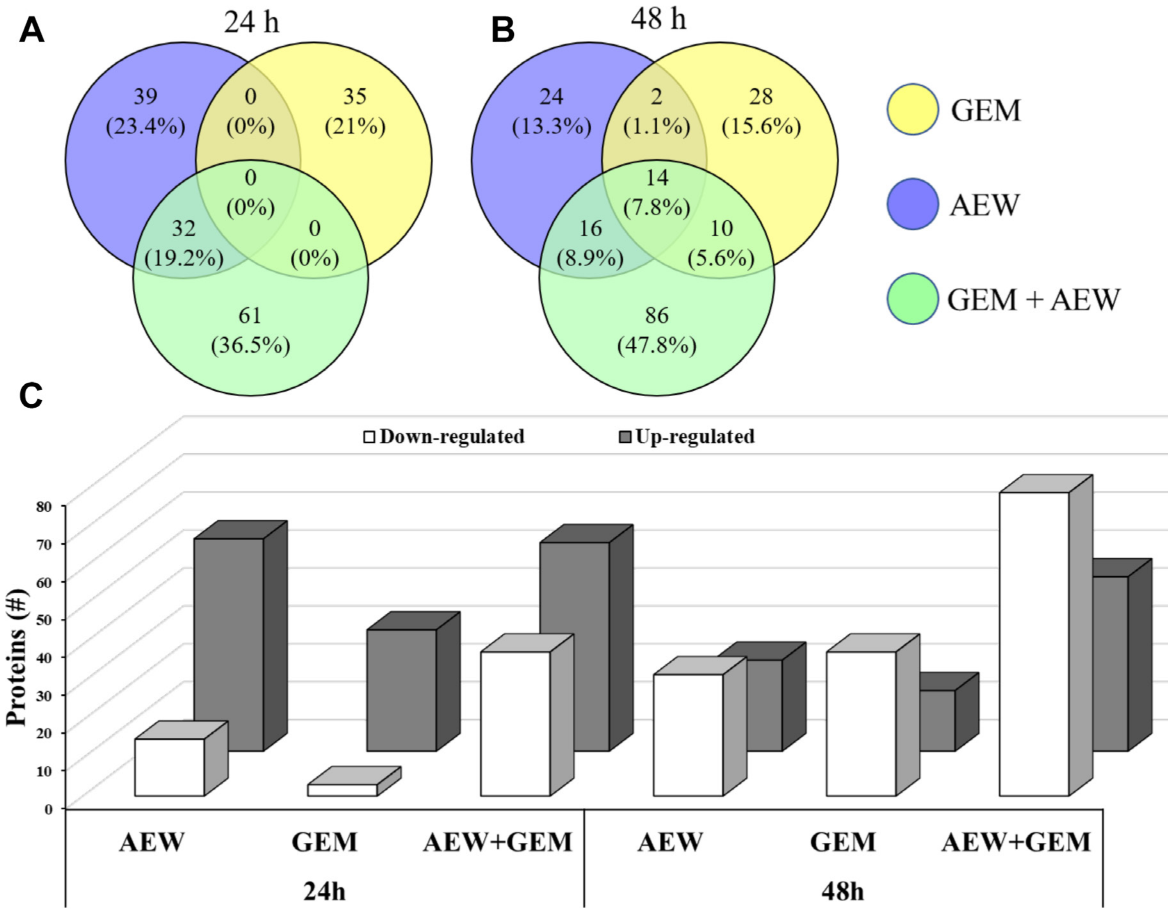

Supplementary Figure 3: Up- and down-regulated differentially expressed proteins as a function of treatment and time. Venn diagrams of differentially expressed proteins lists at 24 (**A**) and 48 (**B**) h following treatment, compared to control (untreated) cells. (A) After 24 h of treatment, a small number of differentially expressed proteins are detected and no synergism in treatment effect is seen, suggesting incomplete effect of the treatment. (B) After 48 h of treatment, a more pronounced effect is seen. Fifty-four, fifty-six and 126 proteins are differentially expressed after treatment with GEM, AEW or both, respectively. There is a 22–29% overlap between GEM and AEW treatments, higher overlap of each treatment effect alone with the combined treatment (GEM+AEW), and fourteen proteins that are common to all treatments compared to control. Moreover, more than twice as much differentially expressed proteins were obtained after the combined treatment than after each treatment alone, suggesting for a synergistic effect of the treatments. (**C**) Bars denote the number of proteins that were up- or down-regulated upon treatment for 24 or 48 h in comparison to controls. As mentioned above, the 24 h treatment may not have been sufficient for detecting a significant effect, which is better presented after 48 h. The increase in the level of differentially expressed proteins in the combined treatment is observed for both up- and down-regulated proteins.

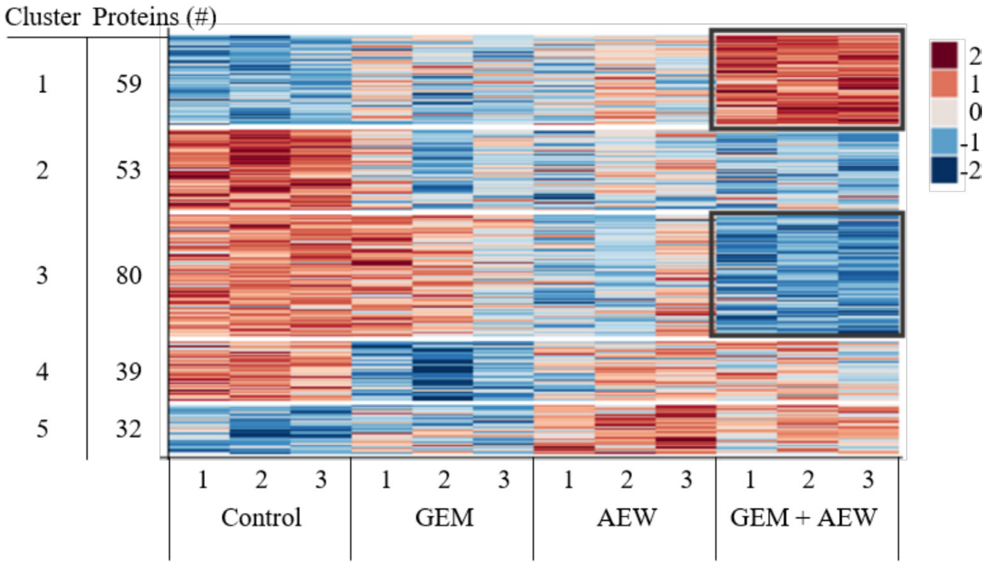

Supplementary Figure 4: K-means clustering analysis of the proteomic data. K-means clustering was used to group proteins with similar profiles. Cluster 1 includes 59 up-regulated proteins and cluster 3 includes 80 down-regulated proteins. The rows represent proteins and the columns represent the treatment in three biological repeats. Up-regulated proteins are marked in red and down-regulated proteins are marked in blue. The proteins included in both clusters are listed in Supplementary Table 2.

Supplementary Table 1: Volcano plot analysis. The table lists proteins identified by Volcano plot analysis. Pink cells represent up-regulated proteins and blue cells represent down-regulated proteins. See Supplementary Table 1

Supplementary Table 2: K-means clustering analysis

Cluster 1 (n = 59) Up-regulated proteins		Cluster 3 (n = 80) Down-regulated proteins	
AFF4	RABGAP1	ARAP1	PRRC2C
AFP	RBBP6	ASNS	PSMC1
AHSG	S100A16	ATG3	PSMC2
BCAS1	SERPINF1	CDV3	PSMC4
BRD3	SH3BGRL	CHORDC1	PSMD2
CALR	SLBP	CLTB	PSPH
CLIC3	STAT1	CLUH	PUM3
CRK	SUCLG2	COG8	PUS7
DDAH1	SYTL2	COMMD9	RAB3D
DEK	TACC1	CRABP2	RBM15
DNM1L	TACC3	CTSB	RBM27
DYNLL1	TPX2	CUL4A	RPL26L1
ECT2	UGDH	DDX21	SCAF8
EPS8L2	USP9X	DDX47	SCYL1
FAM107B	YWHAH	DDX52	SEC23IP
FBLN1		DIS3L2	SFSWAP
GC		DNAAF5	SNF8
GSN		DPF2	SNRPB
HAGH		DRG2	SRPK2
HBA1		EIF3D	SRRM1
HMGN2		EIF3H	TKT
IQGAP3		FAU	TRMT5
ITIH2		GABPA	TSR3
ITIH3		GEMIN5	UBA1
KIAA0101		GNL3	UBE2C
KIAA1033		H1FX	UBE2O
KIF11		HDAC2	UFL1
KIF4A		HERC2	UHRF1
KIFC1		HEXB	USP15
LAP3		HMGCS1	UTP23
LASP1		HUWE1	UTP3
LGALS3		IPO4	VWA9
LIG1		KIF1BP	XPO1
LIMA1		KNOP1	YBX3

LRWD1	LLPH	ZC3H11A
MB	LRPPRC	ZHX2
NA	MYOF	
NDRG1	NAA15	
PLEKHA6	NCDN	
PPFIA1	NOL11	
PPL	PIN4	
PPP1R2	PPP2CB	
PREX1	PPT1	
RAB25	PRDX6	

The table lists the proteins included in Cluster 1 (up- regulated proteins) and Cluster 3 (down-regulated proteins)

Supplementary Table 3: Biological processes associated with up-regulated proteins

High score matches	Biological process	Matched proteins	Matched proteins (Symbols)
16.63	Cell Division	8	KIFC1, KIF11, LIG1, USP9X, TACC1, TPX2, ECT2, TACC3
15.98	Response to Hydrogen Peroxide	4	CRK, HBA1, STAT1, MB
14.47	Cell Cycle	9	KIFC1, KIF11, LIG1, RABGAP1, USP9X, TACC1, TPX2, ECT2, TACC3
13.48	Mitotic Spindle Assembly	3	KIFC1, KIF11, TPX2

The table lists Gene Ontology categories for biological processes that are strongly associated with the up-regulated proteins.

Supplementary Table 4: Biological processes associated with down-regulated proteins

High score matches	Biological process	Matched proteins	Matched proteins (Symbols)
22.38	RRNA Processing	8	DIS3L2, DDX52, DDX47, DDX21, NOL11, UTP23, UTP3, TSR3
14.95	Regulation of Protein Catabolic Process	3	XPOl, SNF8, PSMD2
14.69	Positive Regulation of Proteasomal Protein Catabolic Process	3	PSMC2, PSMC1, PSMC4
14.65	Protein Catabolic Process	4	PSMC2, PSMC1, PSMC4, PPT1
13.70	MRNA Processing	8	GEMIN5, DDX47, RBM27, SNRPB, SCAF8, SRPK2, SRRM1, SFSWAP
13.30	RNA Splicing	7	GEMIN5, DDX47, SNRPB, SCAF8, SRPK2, SRRM1, SFSWAP

The table lists Gene Ontology categories for biological processes that are strongly associated with the down- regulated proteins.

Supplementary Table 5: List of mRNA whose expression differ between LOW and HIGH IGF1R expressing groups. LOW1 and HIGH1 represent levels of IGF1R while LOW2 and HIGH2 represent levels of the named mRNA. See Supplementary Table 5
