# Supplementary material for: Proteomic analysis of combined IGF1 receptor targeted therapy and chemotherapy identifies signatures associated with survival in breast cancer patients
Source: Oncotarget. 2020 Apr 28;11(17):1515–30. doi: 10.18632/oncotarget.27566 (PMC7197451; doi:10.18632/oncotarget.27566)
Supplement: Supplementary file 2 [file oncotarget-11-1515-s002.pdf]

Supplementary Table 1: Volcano plot analysis. The table lists proteins identified by Volcano plot analysis. Pink cells represent up-regulated proteins and blue cells represent down-regulated proteins.

24 hr			48 hr		
**AEW**<br>**88 up**<br>**27 down**	**GEM**<br>**51 up**<br>**4 down**	**AEW+GEM**<br>**95 up**<br>**64 down**	**AEW**<br>**40 up**<br>**51 down**	**GEM**<br>**21 up**<br>**72 down**	**AEW+GEM**<br>**86 up**<br>**134 down**
TIMP3	ANXA6	SERPINC1	DCTN4	ABR	DNM1L
DST	C11orf98	TIMP3	ABR	KRT16	XIAP
TRAP1	NA	PARVB	TXNDC12	GBA	GBA
PRRC1	CAPZB	LCMT1	GBA	COG8	ABR
GSS	NA	TRAP1	ALAD	CC2D1A	PPFIA1
CNOT10	PLEKHG3	IK	CC2D1A	PPFIA1	CDCA5
ESR1	STARD10	DST	SH3GLB1	MAP1LC3B2	RAB25
VPS36	HNRNPD	PGAM5	SNRPD1	GNB1	CC2D1A
CAPN1	ELF2	PRRC1	LGALS3	FBLN1	TRAF4
EEF1B2	HNRNPH1	UNG	THBS1	GPD1L	BCAS1
ABHD14B	ZNF787	CNOT10	S100A10	NECAP1	ZNF706
PCBP2	ALG13	VPS36	GSTZ1	THBS1	AHSG
SLC3A2	ZMYND8	TACC3	EMG1	HBA1	GC
C11orf68	WASH3P	A2M	ZFYVE21	BRD3	PLEKHA6
MVD	NACA	ATP5O	QTRT1	HMGCS1	AFP
UNG	ALYREF	USP39	SELENBP1	YWHAH	TACC1
APPL1	CDK11B	PEPD	MAT2B	ITIH2	LGALS3
AHCYL1	RBM26	GSS	GABARAPL2	FKBP3	TUBB2A
ACAA2	EEF1D	APPL1	LLGL2	SYTL2	FBLN1
NIT2	UBA6	LCP1	HBA1	OSTF1	NA
SEC24A	EPPK1	SVIL	ENOPH1	RPS16	TMSB10
COMT	SBNO1	KIFC1	SUCLG2	CTPS1	ITIH2
ESD	C5orf51	MARCKS	ATP6V0D1	THOC6	PPL
ALDH7A1	UBTF	ZMYND8	OSTF1	H2AFZ	KIAA0101
YPEL5	TARSL2	APEH	PALLD	TARS	IQGAP3
GSTZ1	TCOF1	RBX1	RBM3	MPP6	HBA1

ATP5B	CNOT1	MYO1C	FLNB	NUFIP2	TPX2
RNPEP	TPM1	ENO2	N6AMT1	RBM10	USP9X
STAT2	HNRNPAB	RTF1	FBLN1	SART1	THBS1
SETD1A	FMR1	FN3KRP	COMT	MAT2A	ECT2
PSMD8	FUBP1	COMT	FKBP3	TBL1XR1	CALR
COPE	SPTAN1	RPS27L	RPS16	TYW5	SYTL2
GSE1	PDE4DIP	ATP6V1B2	ITIH2	TRAPPC9	TACC3
LLGL2	HCFC1	CLIC3	PNKP	VTA1	KIAA1033
TSTD1	PFN2	U2AF1	SLK	HIST1H2BJ	DYNLL1
STAT3	AIM1	AHCYL1	CUL7	SMARCD2	HMGN3
FLOT1	PMS2P1	ESD	UBL5	RPL35	KIFC1
XRN1	NUMA1	WIPF2	STARD10	INTS4	SERPINF1
HAGH	G6PD	SLBP	DYNLT1	RWDD1	LAP3
EPM2AIP1	TYW5	AKR1A1	VPS29	GTF2F1	CMC1
RECQL	KIF2A	HZGJ	DDX10	HNRNPH3	SUCLG2
AKR1A1	RAVER1	S100A13	PSAT1	VDAC2	MB
LCP1	LRBA	WNK1	SETD1A	UBLCP1	GANAB
ATP6V1B2	PPP1R9B	SEC24D	SRFBP1	STX16	GSN
CA12	MYO6	CASP7	FRA10AC1	HNRNPUL1	LIMA1
AP1B1	PGP	C21orf33	TSG101	ZNF787	GSTZ1
TRIM26	ZNF316	PREX1	NSF	ABHD14B	POLR2J
FAM98A	DDTL	ALDH16A1	HEXB	VPS50	S100A10
MIF	MAP1LC3B2	CMBL	STX16	MAPK9	KIF11
RBM47	RBM47	CBX5	INTS4	HSP90B1	SELENBP1
SHMT1	DBNL	TSTD1	GEMIN4	RPL7L1	HMGN2
PITPNA	SHTN1	XRCC1	PSMC1	DDX10	STAT1
NUMA1	UNC119B	TXLNG	SUGT1	NUP133	NDRG1
SNRPD3	SMCHD1	STAT2	NUFIP2	FBXL15	LIG1
CMBL	HDLBP	CRYL1	DDX52	DHPS	YWHAH
PRKAR2A		ACAA2	SCAMP3	EPCAM	EPS8L2
GSR		RNF14	PUM3	UTP15	PREX1
PREX1		ARHGAP35	ZNF579	ADSS	ITIH3
HADHA		HSD17B4	CBX1	MEMO1	CLIC3
COPS5		PYGB	PSPH	IMP4	SLBP
DUSP12		AKR7A2	RAB6A	EXOC3	LLGL2
PRPSAP2		PITPNA	GABPA	GSKIP	BRD3
DDAH2		TCF3	LARS	WRNIP1	HAGH

PGD		RECQL	DDX47	OGFOD1	LRWD1
FBP1		ACTN1	WDR36	BUD31	DEK
EIF4A2		RCN1	RPL22L1	BOP1	ATP6V0D1
RPRD1A		NR2C2AP	IRF2BP2	HIST1H2BK	S100A16
XPO4		MACROD1	MAT2A	CALU	QTRT1
VPS35		EHMT1	RRP12	HDGF	AFF4
DIP2B		DBR1	CRABP2	RER1	COMT
ISOC1		ABHD14B	ZC3H11A	RAB6A	RBBP6
ATIC		SYTL2	ADSS	ENO2	UGDH
RPRD1B		CLIC4	TRMT5	PWP1	DDAH1
SVIL		LIMA1	GLB1	CCDC9	RABGAP1
NA		DLD	NOP9	ZMYM2	FAM49B
BLMH		GALK1	ZNF768	NOP9	SH3BGRL
SHOC2		STAT3	ASNS	ERP29	FKBP3
UGGT1		RPRD1B	CTSB	CDK12	OSTF1
RNF113A		HOOK2	IFI30	NSA2	PALLD
ALDH16A1		NA	PPT1	IRF2BPL	KIF4A
THAP11		CMPK1	PFN2	CALCOCO2	FAM107B
SAFB		ACIN1	RRM2B	GANAB	FLNB
TFRC		CKMT1A	KIAA0391	BTF3	LASP1
HMGN5		CHTF8	FLCN	ATP5C1	CRK
PRDX3		YPEL5	SLIRP	HIST2H2AC	PPIC
S100A13		LLGL2	RANBP6	TMEM165	PPP1R2
PACS1		PLCG1	MYO6	MYO6	PFKM
CASP7		NPNT	MBNL3	MZT2B	KDM1A
RTFDC1		TXN	KIF3B	HSDL2	NARFL
PELO		PFKL	ATF7IP	KIAA0391	DDX47
UCK2		CAND1	ZMIZ2	MBNL3	TKT
RABL6		FBP1		KIF3B	KIF1BP
PNMA1		DDAH2		AAR2	BOP1
RPL23		FDPS			DNAAF5
PAF1		FAM98A			VPRBP
FAM83D		DNAJA1			STX16
ADI1		ARMC8			UBA1
IKBKG		EIF3E			EIF3D
KIF2C		SURF2			GNL3
HECTD1		RPL23			H2AFZ

POLR2E		KLC1			RBM27
LLPH		DDX5			USP15
RPL22L1		GRB2			SCYL1
FAIM		ASUN			RAB7A
MED17		GEMIN5			NAA15
NOP16		NOB1			SRRM1
TYMS		DIMT1			XPO1
ZC3H7B		PDCD2			DPF2
DHX16		MYOF			PDCD4
CCDC9		LLPH			CUL4B
RRBP1		PPP6R1			PRDX6
SPATS2L		HECTD1			CHORDC1
EEF2K		IKBKG			TRUB1
RINT1		ESF1			DDX10
ZMIZ2		BZW1			NUDCD1
