# Supplementary material for: Proteomic analysis of combined IGF1 receptor targeted therapy and chemotherapy identifies signatures associated with survival in breast cancer patients
Source: Oncotarget. 2020 Apr 28;11(17):1515–30. doi: 10.18632/oncotarget.27566 (PMC7197451; doi:10.18632/oncotarget.27566)
Supplement: Supplementary file 3 [file oncotarget-11-1515-s003.pdf]

Supplementary Table 5: List of mRNA whose expression differ between LOW and HIGH IGF1R expressing groups. LOW1 and HIGH1 represent levels of IGF1R while LOW2 and HIGH2 represent levels of the named mRNA.

mRNA	HIGH1-HIGH2 (n)	HIGH1-LOW2 (n)	LOW1-HIGH2 (n)	LOW1-LOW2 (n)	mRNA2 p-value	Confidence level
PSMC4	129	443	443	889	1.1353E-07	High
PREX1	309	263	263	1069	1.28071E-07	High
RPL7L1	138	434	434	898	1.89245E-07	High
ZNF787	113	459	459	873	2.36059E-07	High
TRUB1	166	406	406	926	4.4311E-07	High
BOP1	116	456	456	876	6.97803E-07	High
FLNB	348	224	224	1108	7.10104E-07	High
FBLN1	132	440	440	892	8.68619E-07	High
AHSG	178	394	394	938	1.13312E-06	High
MEMO1	82	490	490	842	1.1368E-06	High
SART1	188	384	384	948	1.2111E-06	High
PALLD	133	439	439	893	1.44611E-06	High
PSMC1	103	469	469	863	1.49638E-06	High
MYOF	207	365	365	967	1.62608E-06	High
UTP23	160	412	412	920	1.65659E-06	High
UBE2C	115	457	457	875	1.74035E-06	High
MAT2B	135	437	437	895	1.81918E-06	High
WRNIP1	168	404	404	928	1.87446E-06	High
KIFC1	124	448	448	884	1.98602E-06	High
GTPBP4	88	484	484	848	2.20502E-06	High
PSMD2	130	442	442	890	2.29306E-06	High
PPIC	82	490	490	842	2.43146E-06	High
GNL3	173	399	399	933	2.50346E-06	High
DNM1L	122	450	450	882	2.75986E-06	High
TPX2	109	463	463	869	3.16217E-06	Low
VPRBP	199	373	373	959	3.39763E-06	Low
CALCOCO2	218	354	354	978	3.40121E-06	Low
UBE2O	202	370	370	962	3.70444E-06	Low
LLPH	150	422	422	910	4.30833E-06	Low

COMMD9	146	426	426	906	4.63131E-06	Low
IRF2BP2	158	414	414	918	4.72735E-06	Low
CFL2	152	420	420	912	5.00152E-06	Low
UTP14A	147	425	425	907	5.05413E-06	Low
SH3BGRL	222	350	350	982	5.1078E-06	Low
ARAP1	192	380	380	952	5.58064E-06	Low
SRFBP1	197	375	375	957	5.85228E-06	Low
SNF8	157	415	415	917	6.16116E-06	Low
BTF3	252	320	320	1012	6.2201E-06	Low
ATG3	96	476	476	856	6.36248E-06	Low
COMT	128	444	444	888	6.38563E-06	Low
DDX21	131	441	441	891	6.60696E-06	Low
LGALS3	111	461	461	871	7.11666E-06	Low
DDX10	147	425	425	907	7.16048E-06	Low
PDCD4	205	367	367	965	7.37794E-06	Low
SETD1A	218	354	354	978	7.43278E-06	Low
KDM1A	176	396	396	936	7.7437E-06	Low
NUFIP2	204	368	368	964	8.05359E-06	Low
CALU	90	482	482	850	8.5607E-06	Low
GEMIN5	214	358	358	974	8.71726E-06	Low
UBA1	207	365	365	967	9.41849E-06	Low
QTRT1	255	317	317	1015	9.47348E-06	Low
CUL7	215	357	357	975	9.51856E-06	Low
SLK	162	410	410	922	9.55547E-06	Low
CLTB	128	443	444	889	9.61451E-06	Low
PWP1	102	470	470	862	9.7439E-06	Low
POLR2J	140	432	432	900	9.99375E-06	Low
KIF11	140	432	432	900	1.01106E-05	Low
YWHAH	140	432	432	900	1.01437E-05	Low
ZNF579	217	355	355	977	1.02173E-05	Low
TRMT5	220	352	352	980	1.02746E-05	Low
ASNS	128	444	444	888	1.11339E-05	Low
RBM3	124	448	448	884	1.11948E-05	Low
CC2D1A	212	360	360	972	1.13348E-05	Low
NOL11	157	415	415	917	1.16583E-05	Low
ZFYVE21	156	416	416	916	1.18341E-05	Low
PRDX6	131	441	441	891	1.18409E-05	Low
EIF3D	179	393	393	939	1.19781E-05	Low

SAP30	138	434	434	898	1.2064E-05	Low
BRD3	234	338	338	994	1.21551E-05	Low
GPD1L	248	324	324	1008	1.21819E-05	Low
HDGF	131	441	441	891	1.22394E-05	Low
RANBP6	174	398	398	934	1.2455E-05	Low
EXOC2	243	329	329	1003	1.24616E-05	Low
SRPK2	252	320	320	1012	1.25383E-05	Low
SCAMP3	187	385	385	947	1.26011E-05	Low
PFKM	206	366	366	966	1.26755E-05	Low
HUWE1	224	348	348	984	1.26921E-05	Low
SNRPD1	183	389	389	943	1.28087E-05	Low
CUL5	163	409	409	923	1.28914E-05	Low
KIF3B	236	336	336	996	1.30225E-05	Low
SFSWAP	213	359	359	973	1.32073E-05	Low
ZHX2	176	396	396	936	1.32426E-05	Low
COG8	163	409	409	923	1.32472E-05	Low
KIAA0391	167	405	405	927	1.34441E-05	Low
TXNDC12	180	392	392	940	1.34792E-05	Low
DCTN4	260	312	312	1020	1.36451E-05	Low
THOC6	197	375	375	957	1.38165E-05	Low
UTP3	151	421	421	911	1.38975E-05	Low
HDAC2	99	473	473	859	1.39485E-05	Low
TACC3	141	431	431	901	1.396E-05	Low
ENO2	274	298	298	1034	1.42546E-05	Low
NUDCD1	143	429	429	903	1.43965E-05	Low
ATP5C1	103	469	469	863	1.44021E-05	Low
ERP29	133	439	439	893	1.4413E-05	Low
TUBB2A	163	409	409	923	1.4503E-05	Low
PPFIA1	218	354	354	978	1.45612E-05	Low
RPL26L1	162	410	410	922	1.45825E-05	Low
ZMYM2	237	335	335	997	1.47705E-05	Low
CDV3	168	404	404	928	1.48137E-05	Low
GABPA	185	387	387	945	1.48802E-05	Low
FLCN	171	401	401	931	1.4908E-05	Low
RPL35	152	420	420	912	1.49483E-05	Low
HSP90B1	149	423	423	909	1.49698E-05	Low
IRF2BPL	235	337	337	995	1.4974E-05	Low
MYO6	245	327	327	1005	1.50498E-05	Low

PSMC2	100	472	472	860	1.50524E-05	Low
FRA10AC1	225	347	347	985	1.50977E-05	Low
SUGT1	175	398	397	934	1.51429E-05	Low
NSA2	179	393	393	939	1.51491E-05	Low
RABGAP1	217	355	355	977	1.52016E-05	Low
HNRNPH3	175	397	397	935	1.52457E-05	Low
IFI30	74	498	498	834	1.53572E-05	Low
USP15	188	384	384	948	1.5364E-05	Low
RAB6A	153	419	419	913	1.54081E-05	Low
STARD10	268	304	304	1028	1.54268E-05	Low
S100A16	89	483	483	849	1.54988E-05	Low
SELENBP1	211	361	361	971	1.55724E-05	Low
PIN4	201	371	371	961	1.55902E-05	Low
CALR	106	466	466	866	1.56638E-05	Low
LAP3	98	474	474	858	1.57943E-05	Low
PSAT1	41	531	531	801	1.58445E-05	Low
HMGCS1	136	436	436	896	1.59917E-05	Low
OSTF1	96	476	476	856	1.59972E-05	Low
INTS4	180	392	392	940	1.59993E-05	Low
FKBP3	169	403	403	929	1.60197E-05	Low
RAB3D	158	414	414	918	1.60383E-05	Low
THBS1	140	432	432	900	1.61117E-05	Low
GANAB	171	401	401	931	1.6132E-05	Low
HMGN2	204	368	368	964	1.61573E-05	Low
UGDH	232	340	340	992	1.61607E-05	Low
CLIC3	65	507	507	825	1.62612E-05	Low
UBL5	184	388	388	944	1.62931E-05	Low
AKT1S1	222	350	350	982	1.62991E-05	Low
MAT2A	254	318	318	1014	1.63414E-05	Low
RBBP6	265	307	307	1025	1.63823E-05	Low
RRP12	189	383	383	949	1.64117E-05	Low
LARS	195	377	377	955	1.64125E-05	Low
SYTL2	292	280	280	1052	1.64573E-05	Low
H1FX	237	335	335	997	1.65105E-05	Low
USP9X	145	427	427	905	1.65202E-05	Low
TARS	105	467	467	865	1.65333E-05	Low
ZC3H11A	215	357	357	975	1.65347E-05	Low
TMSB10	86	486	486	846	1.65391E-05	Low

S100A10	65	507	507	825	1.65563E-05	Low
CRABP2	200	372	372	960	1.65748E-05	Low
KIF4A	123	449	449	883	1.66749E-05	Low
STAT1	97	475	475	857	1.66808E-05	Low
ECT2	112	460	460	872	1.66829E-05	Low
ALAD	189	383	383	949	1.67296E-05	Low
EPS8L2	156	416	416	916	1.67441E-05	Low
UBLCP1	116	456	456	876	1.67616E-05	Low
CCDC9	201	371	371	961	1.67685E-05	Low
DIS3L2	207	365	365	967	1.68062E-05	Low
CDK12	58	514	514	818	1.68317E-05	Low
NCDN	255	317	317	1015	1.68346E-05	Low
XPO1	181	391	391	941	1.68551E-05	Low
CTSB	115	457	457	875	1.68867E-05	Low
SEC23IP	235	337	337	995	1.69075E-05	Low
RAB5A	200	372	372	960	1.69606E-05	Low
ADSS	128	444	444	888	1.69623E-05	Low
AFF4	199	373	373	959	1.69651E-05	Low
LRPPRC	185	387	387	945	1.69705E-05	Low
PUS7	147	425	425	907	1.69741E-05	Low
RAB25	188	384	384	948	1.76618E-05	Low
